# Risk of Cancer and Subsequent Mortality in Primary Biliary Cholangitis: A Population-based Cohort Study of 3052 Patients

**DOI:** 10.1016/j.gastha.2023.05.004

**Published:** 2023-06-15

**Authors:** Johanna Schönau, Axel Wester, Jörn M. Schattenberg, Hannes Hagström

**Affiliations:** 1 Department of Internal Medicine I, University Medical Center of the Johannes Gutenberg-University, Mainz, Germany; 2 Department of Medicine, Huddinge, Karolinska Institute, Stockholm, Sweden; 3 Metabolic Liver Research Program, University Medical Center of the Johannes Gutenberg-University, Mainz, Germany; 4 Unit of Hepatology, Department of Upper GI Diseases, Karolinska University Hospital, Stockholm, Sweden

**Keywords:** Cancer, Liver Disease, Hepatocellular Carcinoma, Mortality, Cirrhosis, Population-Based Register Study

## Abstract

**Background and Aims:**

Primary biliary cholangitis (PBC) is a rare cholestatic liver disease. Incident cancer is a concern. Previous studies have described an increase in hepatocellular carcinoma (HCC), but the risk of nonhepatic cancer and cancer risk across subgroups is largely unknown.

**Methods:**

We used the Swedish National Patient Register to identify all patients diagnosed with PBC between 2002 and 2019. Patients were matched for age, sex, and municipality with up to 10 reference individuals from the general population. Incident cancer was recorded from the National Cancer Register. Cox regression was used to investigate the rates of cancer and postcancer mortality, adjusted for potential confounders. The cumulative incidence of cancer was calculated while accounting for the competing risk of death.

**Results:**

At 10 years of follow-up, the cumulative incidence of any cancer in patients with PBC (n = 3052) was 14.3% (95% confidence interval (CI) = 12.8–15.9), compared to 11.8% (95% CI 11.3–12.2) in the reference population (n = 26,792) (adjusted hazard ratio aHR = 1.4, 95% CI = 1.2–1.5). The rate of HCC was particularly high (aHR 30.9; 95% CI = 14.8–64.6). The rate of cancer was higher in patients with cirrhosis (aHR 2.1; 95% CI 1.4–3.0), but similar across categories of age and sex. Increased rates of other cancer subtypes, including gastrointestinal (aHR = 1.5, 95% CI = 1.1–1.9), lung (aHR = 1.5, 95% CI = 1.1–2.2), and lymphoma (aHR = 2.9, 95% CI = 1.9–4.6) were seen. Following a diagnosis of cancer, patients with PBC had higher mortality rates compared to reference individuals (aHR = 1.4, 95% CI = 1.2–1.7). This was mainly driven by HCC (non–HCC-related mortality: aHR = 1.1, 95% CI = 0.9–1.5).

**Conclusion:**

Patients with PBC have a significantly higher risk of HCC compared to matched individuals from the general population, but only a low risk increase of non-HCC cancer.

## Graphical abstract

**Figure:**
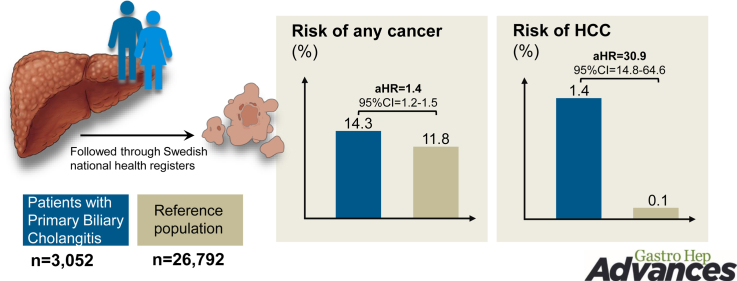

## Background

Primary biliary cholangitis (PBC) is a rare cholestatic liver disease characterised by the progressive destruction of intrahepatic bile ducts, which can progress to cirrhosis and hepatocellular carcinoma (HCC).^1^^,^^2^ In addition, extrahepatic symptoms including fatigue, pruritus, and bone disease affects quality of life and reduces overall survival.^2^ PBC predominantly affects women with a median age of 50 years at onset.^3^ Globally the prevalence of PBC is growing, which might be explained by a true increase in incidence, but also by improved diagnostic tests and increased awareness among physicians. First-line therapy with ursodeoxycholic acid has significantly improved outcomes, but liver transplantation can still be required for patients that develop cirrhosis.^4^^,^^5^

The pathophysiology of PBC is complex and only partly understood. Exposure to environmental factors and genetic predisposition appears to be needed for the progressive inflammation and scarring of intrahepatic bile ducts.^4^ Such exposures might also increase the risk for other diseases. While several studies describe an association of PBC with other autoimmune diseases, the relation with development of especially extrahepatic cancer is still a matter of controversy.^3^^,^^6^ Indeed, the estimation of the true incidence of HCC in chronic liver diseases such as PBC show great disparity, although HCC is the most common primary malignant tumour of the liver in PBC.^3^^,^^7^, ^8^, ^9^ Furthermore, the incidence of HCC in PBC is poorly predicted by prognostic models like the Mayo risk score.^10^ Most previous studies in the field have been small, and data from appropriately sized cohorts is important to derive risk estimates with a high precision to inform clinical care and follow-up.

Liver cirrhosis, regardless of its underlying etiology, increases the risk of HCC.^10^ However, since PBC is rare, it contributes relatively little to the overall incidence of HCC in cirrhosis.^10^^,^^11^ A retrospective study conducted in Sweden showed that alcohol was by far the most important risk factor for HCC development,^11^ confirmed by a review of HCC in the setting of alcohol-related liver disease.^12^

A recent meta-analysis found that patients with PBC have a 19-fold higher risk of developing HCC compared to the general population, which translates to an increased overall risk for cancer.^8^ While several studies have indicated a positive association with extrahepatic cancers such as pancreatic cancer and breast cancer,^13^ the results of the above-mentioned meta-analysis could not detect an increased risk of breast cancer, kidney or colon cancer, as well as other malignancies in PBC.^8^ Previously, a Swedish population-based cohort-study reported a lower overall cancer-related mortality in PBC patients, but an about 20 times increased HCC-related mortality compared to the general population.^1^ However, the study did not examine nonfatal cancer, and did not present absolute risk estimates. In order to inform physicians, patients, and policy-makers, the absolute risk for malignancies is of importance in order to make recommendations for clinical surveillance and screening.^8^ Here, we examined the risk of cancer in patients with PBC to that of matched reference individuals from the general population in a large, population-based cohort study.

## Material and Methods

The DELIVER (DEcoding the epidemiology of LIVER disease in Sweden) is a cohort linking different Swedish population-based registers with information on liver disease diagnoses made between 1964 and 2020. By matching every included patient with up to 10 reference individuals from the general population for age, sex, municipality and calendar year of first liver disease diagnosis, the database allows researchers to explore the clinical course of several liver diseases and their risk of comorbidities and complications.^14^

Herein, we used the DELIVER cohort to conduct a national, population-based, and matched cohort study. We used the National Patient Register^15^ to identify all Swedish individuals with a diagnosis of PBC as a primary or contributing diagnosis at any time between January 1, 2002 and October 31, 2019. This register includes data on inpatient care since 1964 and specialty outpatient care since 2001. Since most patients with PBC are mainly diagnosed in outpatient care, we decided to not start the study before 2001 to reduce selection bias related to different inclusion periods. To exclude patients with a known PBC diagnosis, the start of the study was set to January 1, 2002, and the data from 2001 were used as a look-back period. That is, we only included patients with a new diagnosis of PBC to reduce the risk of survival bias. The National Patient Register has been externally validated and found to be highly accurate.^15^

### Study population

**Figure 1:**
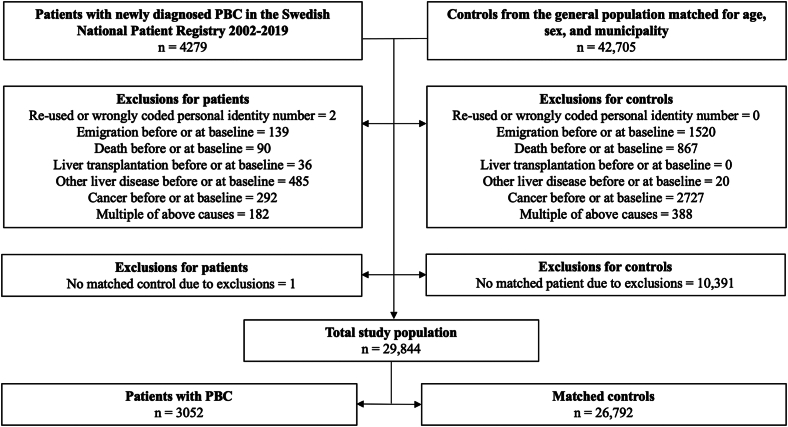
Flowchart of study exclusions.

We defined PBC as an International Classification of Disease (ICD-10) code of K74.3. We did not have data to differentiate between morphological disease stages other than presence of cirrhosis (see below: follow up and outcomes). We deliberately did not include individuals with an ICD-10 coding for “biliary cirrhosis, unspecified” (K74.5) since the nomenclature was adjusted from “cirrhosis” to “cholangitis” in 2014 as only few patients actually develop cirrhosis.^1^^,^^16^ Also, the sensitivity of the National Patient Register was high for most diseases.^15^ A comprehensive list of all ICD codes used to define exposure and outcomes is presented in the Tables A1, A2 and A3. Patients with a previous liver transplantation before or at baseline were excluded, as well as patients with ICD-coding for other liver diseases than PBC or patients with any diagnosed cancer (except nonmelanoma skin cancer) before or at baseline (Figure 1). Each individual with PBC was then compared with up to 10 matched reference individuals from the DELIVER dataset, applying identical inclusion and exclusion criteria.

### Follow-up and outcomes

The start of follow-up (baseline) was 90 days after the first time point of ICD coding for PBC in the National Patient Register. Reference individuals started the follow-up at the same date as their matched patients. The time lag of 90 days was used to identify patients who already had cancer at PBC diagnosis, but where the malignancy was first detected due to examinations related to the PBC diagnosis. Thus, follow-up was from April 1, 2002 until December 31, 2019.

Incident cancers were defined by ICD-10 codes in the Swedish Cancer Register^16^ (Table A2). Cancers in specific organs were summarized into organ-systems, for example: esophagus, stomach, small intestine, and colorectal cancer were categorized as gastrointestinal cancer. A time-to-event analysis was performed for the primary outcome “any cancer”. We only considered the first cancer after baseline. As cancer risk might differ for different categories of patients, we investigated the primary outcome stratified on the following parameters: sex, age group (<50, 50–65 and >65 year old), and presence of cirrhosis. Cirrhosis was defined by coding for cirrhosis, esophageal varices, ascites, or hepatorenal syndrome. For this subgroup analysis, we compared each patient with PBC and the particular parameter with up to 10 general population individuals with the same age, sex, and municipality.

Secondary outcomes included specific cancer sites, defined as gastrointestinal; hepatocellular; pancreaticobiliary; lung; breast; genitourinary; lymphoma; or hematological cancer except lymphoma (definitions in Table A2).

Separately, we investigated all-cause, cancer-related, and non–cancer-related mortality within 1 year and 5 years *after* any cancer, comparing this to reference individuals who were also diagnosed with cancer. Mortality outcomes were ascertained from the Total Population Register.^17^^,^^18^ Causes of death (cancer-related or non–cancer-related) were defined by ICD-codes from the Cause of Death Register^19^ according to Table A3. For this analysis, follow-up started at the date of the diagnosis of cancer during the study period.

End of follow-up was the occurrence of the first diagnosed cancer or a censoring event (emigration from Sweden, end of the study period (December 31, 2019), liver transplantation or death for the primary analysis. For the mortality analysis after cancer diagnosis, end of follow-up was mortality or a censoring event (emigration from Sweden, end of the study period (December 31, 2019) or liver transplantation). Data on follow-up time were extracted from the Total Population Register which contains demographic data including emigration and date of death.^15^

### Variables at baseline

Parameters collected at the index date included sex, age and country of birth. We further recorded established diagnoses at or before baseline for several comorbidities: cardiovascular disease (CVD), diabetes type 1 and 2, inflammatory bowel disease (IBD), chronic obstructive pulmonary disease (COPD), rheumatic disease, and cirrhosis. The definitions of these were based on ICD codes listed in Table A1.

### Statistical analysis

Rates of cancer per 1000 person-years and the cumulative incidence (CI) of any cancer at 1, 5 and 10 years and after full follow-up were calculated. For the cumulative incidences, we accounted for the competing risk of death. Descriptive statistics for continuous variables were expressed as medians and interquartile ranges (IQR), and categorical variables were presented as absolute numbers and percentages. Cox regression was used to assess the rate of cancer in patients with PBC compared to their reference individuals. We considered 2 separate models. The first model accounted for the matching factors (age, sex and municipality). In the adjusted model, the following covariates were included as possible confounders: CVD, diabetes, IBD, rheumatic disease, COPD and education (<10, 10–12, >12 years).

Cox regression was also used to compare all-cause, cancer-related and non–cancer-related mortality between patients with PBC and reference individuals after 1 and after 5 years. This analysis was done after the first date of the diagnosis of any cancer and consequently only includes patients and reference individuals who had been diagnosed with cancer during follow-up. The mortality rate was adjusted for the above-mentioned covariates as well. Furthermore, mortality rates after non-HCC cancer during follow-up were conducted. For this purpose, patients with PBC and non-HCC cancer were compared with reference individuals that also developed cancer, adjusted for age, sex, municipality, and calendar year of first liver disease diagnosis.

The cumulative incidence for both main and secondary outcomes were presented in cumulative incidence curves. All-cause mortality was presented in Kaplan–Meier curves. Analyses were performed using Stata V.17.0.

### Ethical considerations

The study was conducted in accordance with the Helsinki Declaration of 1975, as revised in 1983 and approved by the regional Ethics Committee, Stockholm, Sweden (registry nr 2017/1019-31/1).

## Results

### Baseline characteristics of the PBC and control cohorts

We identified a total of 3052 PBC patients between 2002 and 2019. These were matched with 26,792 reference individuals. Table 1 lists baseline characteristics of the included individuals. Based on matching for age, sex and municipality, 85% of included cases were females. The median age at baseline was 64 (IQR: 55–73) years in patients with PBC compared to 63 years in general population individuals (IQR: 54–72). Further characteristics on origin, marital status and education are shown in Table 1. At baseline, all monitored comorbidities were more common in patients with PBC (Table 1). Interaction tests confirmed a statistically significant difference between patients with PBC and reference individuals. A total of 27.8% of patients with PBC and 17.1% of the control individuals had a diagnosis of CVD, COPD in 3.3% vs 1.8% and cirrhosis was present in 10.6% of patients with PBC but only in 1 person of the reference individuals (Table 1).

**Table 1:** Baseline Characteristics of the PBC Population and Reference Individuals

	PBC population	Reference population	*P*-value
Included persons, n	3052	26,792	
Follow-up (y) (median, IQR)	5.5 (2.3–10.3)	7.0 (3.3–12.1)	<.001
Sex, women n (%)	2606 (85.4)	22,984 (85.8)	.549
Age at baseline, y (median, IQR)	64 (55–73)	63 (54–72)	.016
Period of inclusion n (%)			.347
2002–2007	1124 (36.8)	10,183 (38.0)	
2008–2013	891 (29.2)	7815 (29.2)	
2014–2019	1037 (34.0)	8794 (32.8)	
Country of birth n (%)			.291
Nordic	2740 (89.8)	24,213 (90.4)	
Other	312 (10.2)	2579 (9.6)	
Education, n (%)			<.001
<10 y	994 (33.0)	7787 (29.4)	
10–12 y	1329 (44.1)	11,132 (42.0)	
>12 y	691 (22.9)	7564 (28.6)	
Married, n (%)	1586 (52.0)	14,165 (52.9)	.343
Comorbidity at / before baseline n (%)			
CVD	847 (27.8)	4581 (17.1)	<.001
Diabetes	270 (8.9)	1259 (4.7)	<.001
IBD	77 (2.5)	242 (0.9)	<.001
COPD	102 (3.3)	489 (1.8)	<.001
Rheumatic disease	313 (10.3)	752 (2.8)	<.001
Cirrhosis	323 (10.6)	1 (0.0)	<.001

PBC, primary biliary cholangitis; CVD, cardiovascular disease; IBD, inflammatory bowel disease; COPD, chronic obstructive pulmonary disease.

### Rate and risk of cancer

**Figure 2:**
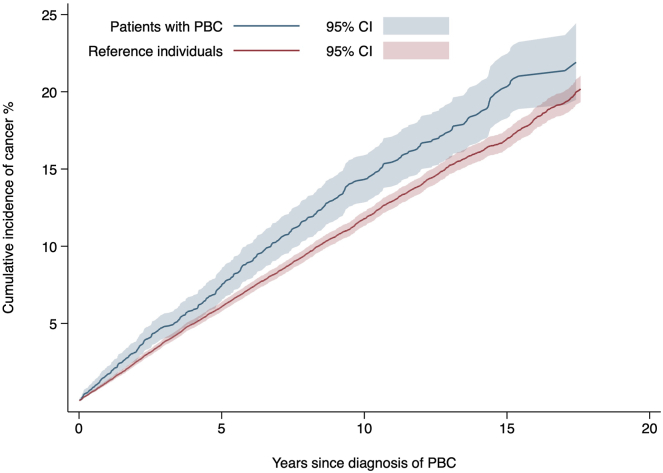
Cumulative incidence of cancer in patients with primary biliary cholangitis (PBC) and matched reference individuals.

Table 2 presents cancer outcomes in patients with PBC and in reference individuals by cancer site. Over a median follow-up of 5.5 years, 374 incident cases of any cancer were diagnosed among patients with PBC (18.4/1000 person-years (PY), 95% confidence interval (CI) = 16.7–20.4). In the matched reference group 2931 newly diagnosed cancer cases were detected (13.9/1000 PY, 95% CI = 13.4–14.4). This difference translated to a hazard ratio (HR) of 1.4 (95% CI = 1.2–1.5) for any incident cancer in PBC. After adjustment for CVD, diabetes, IBD, rheumatic disease, COPD, and education, patients with PBC still had a 1.4-fold higher rate of any cancer compared to reference individuals (adjusted HR (aHR): 1.4; 95% CI = 1.2–1.5) (Figure 2).

**Table 2:** Rate of Cancer in Patients With PBC and Reference Individuals

	Patients with PBC	Reference individuals	Patients with PBC	Reference individuals	Unadjusted HR (95% CI)^a^	Adjusted HR (95% CI)^b^
	Events, n (%)	Events, n (%)	Incidence rate/1000 PY (95% CI)	Incidence rate/1000 PY (95% CI)		
Any cancer	374 (12.3)	2931 (10.9)	18.4 (16.7–20.4)	13.9 (13.4–14.4)	1.4 (1.2–1.5)	1.4 (1.2–1.5)
Non-HCC cancer	334 (10.9)	2910 (10.9)	16.4 (14.8–18.3)	13.8 (13.3–14.3)	1.2 (1.1–1.4)	1.2 (1.1–1.4)
Gastrointestinal cancer	74 (2.4)	543 (2.0)	3.5 (2.8–4.4)	2.5 (2.3–2.7)	1.5 (1.2–1.9)	1.5 (1.1–1.9)
Esophagus	1 (0.0)	19 (0.1)	0.0 (0.0–0.3)	0.1 (0.1–0.1)	0.6 (0.1–4.2)	0.3 (0.0–3.1)
Stomach	7 (0.2)	41 (0.2)	0.3 (0.2–0.7)	0.2 (0.1–0.3)	1.9 (0.8–4.4)	2.3 (1.0–5.5)
Small intestine	6 (0.2)	19 (0.1)	0.3 (0.1–0.6)	0.1 (0.1–0.1)	4.0 (1.5–10.6)	3.8 (1.3–11.4)
Colorectal	61 (2.0)	466 (1.7)	2.9 (2.2–3.7)	2.1 (1.9–2.3)	1.4 (1.1–1.9)	1.4 (1.1–1.9)
Hepatocellular carcinoma	44 (1.4)	22 (0.1)	2.0 (1.5–2.8)	0.1 (0.1–0.2)	23.1 (12.8–41.7)	30.9 (14.8–64.6)
Pancreaticobiliary cancer	31 (1.0)	278 (1.0)	1.4 (1.0–2.1)	1.3 (1.1–1.4)	1.2 (0.8–1.8)	1.2 (0.8–1.8)
Pancreas	6 (0.2)	86 (0.3)	0.3 (0.1–0.6)	0.4 (0.3–0.5)	0.7 (0.3–1.7)	0.7 (0.3–1.6)
Biliary tract	25 (0.8)	192 (0.7)	1.2 (0.8–1.7)	0.9 (0.8–1.0)	1.5 (1.0–2.2)	1.5 (1.0–2.3)
Lung cancer	41 (1.3)	257 (1.0)	1.9 (1.4–2.6)	1.2 (1.0–1.3)	1.7 (1.2–2.4)	1.5 (1.1–2.2)
Breast cancer	70 (2.3)	673 (2.5)	3.3 (2.6–4.2)	3.1 (2.8–3.3)	1.1 (0.8–1.4)	1.1 (0.8–1.4)
Genitourinary cancer	58 (1.9)	628 (2.3)	2.7 (2.1–3.5)	2.9 (2.6–3.1)	1.0 (0.7–1.3)	1.0 (0.7–1.3)
Cervix	14 (0.5)	95 (0.4)	0.7 (0.4–1.1)	0.4 (0.3–0.5)	1.4 (0.8–2.5)	1.3 (0.7–2.4)
Uterus	10 (0.3)	166 (0.6)	0.5 (0.3–0.9)	0.7 (0.6–0.9)	0.6 (0.3–1.2)	0.6 (0.3–1.2)
Ovary	4 (0.1)	75 (0.3)	0.2 (0.1–0.5)	0.3 (0.3–0.4)	0.6 (0.2–1.5)	0.6 (0.2–1.8)
Prostate	15 (0.5)	180 (0.7)	0.7 (0.4–1.2)	0.8 (0.7–0.9)	0.9 (0.5–1.6)	1.0 (0.6–1.7)
Kidney	1 (0.0)	8 (0.0)	0.0 (0.0–0.3)	0.0 (0.0–0.1)	1.4 (0.2–11.3)	1.5 (0.2–14.9)
Urine bladder	15 (0.5)	112 (0.4)	0.7 (0.2–1.2)	0.5 (0.4–0.6)	1.40(0.8–2.4)	1.3 (0.7–2.3)
Lymphoma	28 (0.9)	101 (0.4)	1.3 (0.9–1.9)	0.5 (0.4–0.6)	3.0 (1.9–4.6)	2.9 (1.9–4.6)
Hematological cancer except lymphoma	10 (0.3)	101 (0.4)	0.5 (0.3–0.9)	0.5 (0.4–0.6)	1.1 (0.6–2.2)	1.2 (0.6–2.3)
Myeloma	6 (0.2)	46 (0.2)	0.3 (0.1–0.6)	0.2 (0.2–0.3)	1.4 (0.6–3.5)	1.5 (0.6–3.8)
Leukemia and other hematological cancers	4 (0.1)	55 (0.2)	0.2 (0.1–0.5)	0.2 (0.2–0.3)	0.8 (0.3–2.3)	0.9 (0.3–2.6)

PBC, primary biliary cholangitis; PY, Person years; HR, hazard ratio; HCC, hepatocellular carcinoma.

a Analyzed within age, sex, and municipality-matched strata.

b Analyzed within age, sex, and municipality-matched strata and further adjusted for CVD, diabetes; IBD, rheumatic disease; COPD, and education.

The incidence of HCC (44 events; 1.4%) was higher in patients with PBC compared to the control group (22 events; 0.1%). This translated into a 30-fold increase in the rate of HCC (aHR = 30.9; 95% CI = 15–65). When conducting further subgroup analyses by only looking at non-HCC cancer, PBC still remained associated with a slightly increased risk (aHR = 1.21; 95% CI = 1.1–1.4). For other cancers, the analysis according to cancer site is shown in Table 2. PBC patients showed a nearly 1.5-fold increase of lung cancer (aHR = 1.5; 95% CI = 1.1–2.2) and a similar increase in gastrointestinal cancer (aHR = 1.5; 95% CI = 1.1–1.9). Within the group of gastrointestinal cancer, the greatest difference in PBC compared to reference individuals was seen for stomach (aHR of 2.3; 95% CI = 1.0–5.7) and small intestine cancer (aHR: 3.8 95% CI = 1.3–11.4). No difference was observed for pancreaticobiliary cancer with 6 cases (0.2%) of pancreatic cancer in the PBC group and 86 cases (0.3%) in the control group, and 25 cases (0.8) vs 192 (0.7) cases of biliary cancer in PBC and control, respectively. Likewise, no difference was seen for breast and genitourinary cancer (Table 2). A higher number of incident lymphoma was observed in patients with PBC with 1.3 /1000 PY in PBC vs 0.5 /1000 PY in reference individuals (aHR 2.91; 95% CI = 1.8–4.6). The rate of other haematological cancers including myeloma or leukaemia was not increased in PBC (Table 2). Table A4 summarizes the rates of any cancer in prespecified subgroups, comparing patients with PBC to their matched reference individuals. We conducted interaction tests in order to emphasize statistically significant differences between the groups.

The impact of PBC on incident cancer was similar in women (aHR = 1.4; 95% CI = 1.2–1.5) and men (aHR = 1.3; 95% CI = 0.9–1.7) (p_interaction_ = 0.453). The rates of cancer increased with age, but there was no significant difference in cancer rates depending on age between patients with PBC and matched individuals (p_interaction_ = 0.328). The age group >65 years showed the highest incidence rate per 1000 PY in both groups (patients with PBC: 23.2/1000 PY; matched references: 18.8/1000 PY). When restricted to only those with PBC who had a diagnosis of cirrhosis, the rate of any cancer was more than 2 times higher in the PBC cohort compared to reference individuals (aHR = 2.1; 95% CI = 1.4–3.0). PBC patients without a diagnosis of cirrhosis had a 1.3-fold increase of cancer (95% CI = 1.2–1.5) compared to their matched reference individuals.

Table 3 summarizes the cumulative incidence of any cancer during follow up at 1, 5, 10, and the maximal follow-up of 18 years. Patients with PBC were at a higher risk for cancer at all time points during follow-up. At 10 years, the 2 groups showed the greatest difference of cumulative incidence of cancer (patients with PBC: 14.3%; matched reference individuals: 11.8%). Considering subgroups, women with PBC showed a higher 18-year cumulative incidence (22.2%) than their female matched references (19.6%). The estimated cumulative incidence for men after 18 years of follow-up was higher in the control group (23.9%; patients with PBC: 20.0%). The age group 50–65 years had more cancer diagnoses compared to participants <50 years old in both groups and at all time points, the values reached their peak at full follow-up (18 years) with a cumulative incidence of 26.4% in patients with PBC and 21.8% in reference individuals. The cumulative incidence dropped again in the age group >65 years at full follow-up (18.8%; 21.2%), which can be explained by accompanying illnesses other than cancer that influenced the survival of the individuals. The rates of cancer were higher in patients with cirrhosis (aHR = 2.1; 95% CI = 1.4–3.9) than in patients without cirrhosis (aHR = 1.3; 95% CI = 1.2–1.5). During the first years after baseline, patients with cirrhosis had higher risks of cancer, as well as patients without cirrhosis. The high competing risk of death led to a somewhat lower cumulative incidence of cancer for patients with PBC at the full follow-up in the cirrhosis group.

**Table 3:** Cumulative Incidence of Any Cancer During Follow-up

Group	Patients with PBC	Reference individuals	Patients with PBC	Reference individuals	Patients with PBC	Reference individuals	Patients with PBC	Reference individuals
Cumulative incidence (%), (95% CI)	At 1 y	At 1 y	At 5 y	At 5 y	At 10 y	At 10 y	At 18 y	At 18 y
Overall	1.7 (1.3–2.2)	1.2 (1.1–1.4)	7.5 (6.5–8.5)	6.1 (5.8–6.4)	14.3 (12.8–15.9)	11.8 (11.3–12.2)	21.9 (19.5–24.4)	20.2 (19.3–21.0)
By subgroup								
Sex								
Women	1.5 (1.1–2.0)	1.2 (1.1–1.4)	7.4 (6.3–8.5)	5.8 (5.5–6.2)	14.2 (12.6–15.9)	11.3 (10.8–11.8)	22.2 (19.5–25.0)	19.6 (18.7–20.5)
Men	2.8 (1.5–4.6)	1.3 (1.0–1.7)	8.1 (5.6–11.2)	7.6 (6.7–8.6)	15.2 (11.2–19.7)	15.0 (13.6–16.4)	20.0 (14.6–26.0)	23.9 (21.2–26.6)
Age group								
<50	0.5 (0.1–1.6)	0.7 (0.4–1.0)	3.7 (2.0–6.0)	2.6 (2.1–3.2)	9.0 (5.9–13.0)	5.5 (4.7–6.4)	16.9 (11.3–23.6)	11.2 (9.1–13.5)
50–65	2.2 (1.4–3.1)	0.9 (0.8–1.1)	7.4 (5.9–9.1)	5.1 (4.7–5.6)	15.9 (13.5–18.5)	11.4 (10.7–12.1)	26.4 (22.2–30.7)	21.8 (20.5–23.2)
>65	1.7 (1.1–2.5)	1.7 (1.5–2.0)	8.7 (7.2–10.5)	8.3 (7.8–8.9)	14.5 (12.4–16.8)	14.4 (13.6–15.2)	18.8 (16.0–21.8)	21.2 (19.9–22.5)
Disease severity^a^								
Cirrhosis	2.6 (1.2–4.8)	1.3 (0.9–1.8)	8.3 (5.3–12.0)	7.4 (6.3–8.5)	14.5 (10.2–19.6)	12.8 (11.3–14.3)	17.4 (11.8–23.8)	18.9 (16.7–21.2)
No cirrhosis	1.6 (1.2–2.1)	1.2 (1.1–1.4)	7.4 (6.3–8.5)	5.9 (5.6–6.3)	14.3 (12.7–16.0)	11.7 (11.2–12.2)	22.4 (19.8–25.1)	20.3 (19.4–21.2)

PBC, primary biliary cholangitis.

a Disease severity (cirrhosis/no cirrhosis) only refers to patients with PBC.

### Cancer-related mortality in PBC and reference individuals

**Figure 3:**
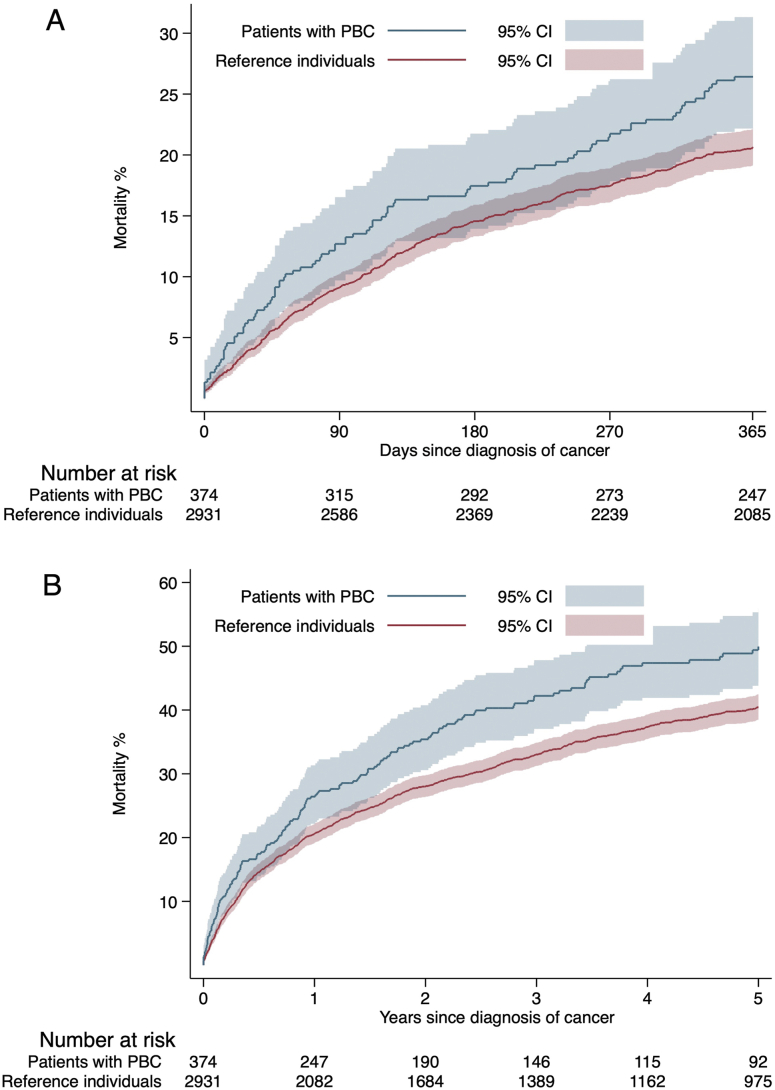
Kaplan-Meier failure functions of 1-year (A) and 5-year (B) all-cause mortality after cancer in patients with PBC and matched reference individuals.

Patients with PBC were at increased risk of death following a diagnosis of cancer compared to the reference individuals (Figure 3). This was seen both for cancer-related as well as non–cancer-related mortality (Table 4). We observed an approximately 1.4-fold relative increase in all-cause mortality in patients with PBC after 1 and 5 years in the fully adjusted model. At 5 years, PBC patients had an overall mortality rate of 173.6/1000 PY, compared to an overall mortality rate of 124.5/1000 PY in reference individuals. This was mostly attributed to cancer-related mortality. The increase in cancer-related mortality was 138.7/1000 PY in patients with PBC, vs 108.2/1000 PY in reference individuals. In non–cancer-related mortality, incidence rates/1000 PY were 34.9 in patients with PBC, compared to 16.3 in reference individuals. The 1-year mortality rates demonstrated an increased risk of death in the PBC group with an aHR of 1.3 (95% CI = 1.1–1.7) for cancer-related death and an aHR of 2.2 (95% CI = 1.1–4.1) for noncancer death. Five-year mortality rates showed similar results with an aHR of 1.3 (95% CI = 1.1–1.6) for cancer-related death and 2.3 (1.5–3.4) for noncancer death.

**Table 4:** Mortality Rate in Patients With PBC and Reference Individuals Diagnosed With Cancer During Follow-up

	Patients with PBC	Reference individuals	Patients with PBC	Reference individuals	Unadjusted HR (95% CI)	Adjusted HR (95% CI)
	Events, n (%)	Events, n (%)	Incidence rate/1000 PY (95% CI)	Incidence rate/1000 PY (95% CI)		
1-y mortality						
All-cause	95 (25.4)	581 (19.8)	321.5 (263.0–393.1)	241.0 (222.2–261.5)	1.5 (1.2–1.8)	1.4 (1.1–1.8)
Cancer-related	83 (22.2)	533 (18.2)	280.9 (226.5–348.3)	221.1 (203.1–240.7)	1.4 (1.1–1.8)	1.3 (1.1–1.7)
Non-cancer-related	12 (3.2)	48 (1.6)	40.6 (23.1–71.5)	19.9 (15.0–26.4)	2.3 (1.2–4.4)	2.2 (1.1–4.1)
5-y mortality						
All-cause	159 (42.5)	1015 (34.6)	173.6 (148.6–202.8)	124.5 (117.1–132.4)	1.5 (1.3–1.8)	1.4 (1.2–1.7)
Cancer-related	127 (34.0)	882 (30.1)	138.7 (116.6–165.0)	108.2 (101.3–115.6)	1.4 (1.1–1.6)	1.3 (1.1–1.6)
Non–cancer-related	32 (8.6)	133 (4.5)	34.9 (24.7–49.4)	16.3 (13.8–19.3)	2.7 (1.8–3.9)	2.3 (1.5–3.4)

PBC, primary biliary cholangitis; PY, Person years; HR, hazard ratio.

Further analyses showed the mortality rates after non-HCC cancer, with no significant differences between PBC and reference individuals. As shown in Table A5, patients with PBC had a 1.2-fold rate of all-cause death at 1 year after being diagnosed with non-HCC cancer, compared to the reference group (5 years: 1.3-fold rate). PBC patients had a mortality rate of 232.3/1000 PY 1 year after non-HCC cancer, compared to 218.5/1000 PY for reference individuals after non-HCC cancer. These numbers translated into an aHR of 1.1 (95% CI = 0.9–1.5). The 5-year mortality showed equal results (aHR = 1.1 (95% CI = 0.9–1.4). Non–HCC-cancer related mortality showed greater differences in patients with PBC compared to matched individuals, with an aHR of 2.0 (95% CI = 1.0–4.1) at 1 year, and an aHR of 2.2 (95% CI = 1.4–3.3) at 5 years.

## Discussion

Here, we quantify the risk of cancer as well as mortality following a cancer diagnosis in patients with PBC in a large population-based cohort study. We observed a 1.4-fold increased incidence rate of any cancer in patients diagnosed with PBC, compared to reference individuals. The risk was particularly high for HCC with an adjusted HR of 30.9. Cancer-related, as well as non–cancer-related mortality rates were higher in patients with PBC compared to reference individuals following a cancer diagnosis, resulting in an approximately 1.4-fold increased all-cause mortality. The increased risk of death after cancer in PBC was mostly driven by a higher proportion of HCC, that carries a more dismal prognosis compared to other cancers.

The current analysis included 3052 patients with an ICD-10 coded diagnosis of PBC after 2002 derived from the Swedish National Patient Register. In a previous analysis, Marschall and colleagues explored the same register for overall mortality in PBC and included cases from 1987 until 2014.^1^ It is important to note that the protocol of the Swedish Patient Register changed in 2001 and included also outpatients from 2001 onwards, while historically only hospitalizations were registered before 2001.^1^ Additionally, we examined also nonfatal cancer, and only included patients with a new diagnosis of PBC, why our risk estimates might be more accurate due to less problems with selection and survival bias.

The current study expands the available literature and underlines the increased risk of cancer in patients with PBC that was seen in the majority, but not all studies published previously.^20^ In a meta-analysis that included 17 studies with 16,300 PBC patients, the pooled relative risk of any cancer was 1.55 (95% CI = 1.3–1.8)^8^. This study observed a pooled relative risk for HCC of 18.8 (95% CI = 10.8–26.8) but did not find an increase in other cancer subtypes.^8^ In this study, an increased rate of gastric and stomach cancer was detectable only for male PBC patients.^8^ Our study reinforces the increased risk of HCC and expands the findings by showing a relevant increase in lymphoma and lung cancer. Beyond HCC, the strongest association was found for gastrointestinal cancer. An increased risk for pancreatic cancer as previously seen in patients from the Veterans Affairs system—which is by default enriched for male cases—by Landgren and colleagues,^21^ was not confirmed here, although the small number of outcomes of this rare cancer precludes definite conclusions.

A novel finding of the current analysis was the increased risk of lymphoma. This has not been previously described, and further studies are needed to examine the association in higher detail, such as the effect of any immunomodulatory treatments in PBC.

The risk of cancer was higher than in the reference population across all investigated subgroups and was impacted by age. As described previously, males had a higher risk of cancer compared to females in both groups, which has been attributed to genetic, hormonal, and behavioral differences.^22^ Interaction tests indicated that rates of cancer in patients with PBC were similar to those in reference individuals, independent of age and sex.

A central finding of our analysis was an increase in cancer-related as well as non–cancer-related mortality following a cancer diagnosis in patients with PBC. These findings align with a smaller study from northern England.^6^ Non–cancer-related mortality even exceeded the risk of cancer-related death in PBC compared to matched reference individuals mostly related to digestive, cardiovascular, and respiratory as the most frequent causes of death as previously described.^1^ In the present cohort study, we additionally demonstrated that the finding of an increased cancer-related death after a diagnosis of cancer in PBC patients was mostly driven by HCC. Patients with PBC with other cancers than HCC had similar survival as reference individuals following a cancer diagnosis.

The following strengths and limitations are acknowledged. The analysis is based on large, prospectively maintained national registers. For the purpose of the study patients diagnosed between 2002 and 2019 were included representing a modern cohort of patients with PBC. Importantly, we could compare outcomes with 26,792 matched individuals from the general population. The link of the Swedish national registers with national death certificates allowed to assess overall mortality and specific causes. Importantly, very few cases were lost during the long follow-up period. We were able to analyze all data with age, sex and municipality matched strata, and further adjusted for comorbidities including COPD as a proxy for smoking. We did not have data on some parameters that might further explain the association between PBC and cancer. For instance, hepatic steatosis and more granular stages of fibrosis. However, we adjusted for DM type 1 and 2, with DM type 2 being indicative for a high BMI since they are often associated. Future studies should examine to what degree such risk factors may affect the found association. Another limitation of the study is the lack of linked laboratory data or clinical data in higher detail such as data on drug prescriptions, and thus the inability to assess liver function or treatment response in relation to mortality and cancer. Also, this required to rely on ICD-10 codes rather than conforming individual diagnosis according to EASL criteria for PBC.

## Conclusion

In conclusion, this large nationwide population-based cohort study found a 1.4-fold higher rate of cancer in patients with PBC compared to reference individuals from the general population, with the highest risk estimates found for HCC. However, also after excluding incident HCC, patients with PBC had a higher cancer rate. We found other specific cancer subtypes that were more common in PBC, including gastrointestinal cancer and lymphoma. Additionally, following a cancer diagnosis, patients with PBC have higher mortality compared to matched reference individuals also with cancer—however, this was highly driven by a higher proportion of the patients with PBC having HCC, which carries a more dismal prognosis than many other cancers. The risk increase of non-HCC cancer was low, suggesting that patients with PBC should not be specifically investigated for such cancers.

## Supplementary Materials

Tables A1–A5
