# Supplementary material for: Risk of Cancer and Subsequent Mortality in Primary Biliary Cholangitis: A Population-based Cohort Study of 3052 Patients
Source: Gastro Hep Adv. 2023 Jun 15;2(7):879–88. doi: 10.1016/j.gastha.2023.05.004 (PMC11307889; doi:10.1016/j.gastha.2023.05.004)
Supplement: Supplementary file 1 — Tables A1–A5 [file mmc1.docx]

# SUPPLEMENTAL MATERIAL

**Risk of cancer and subsequent mortality in Primary Biliary Cholangitis: A population-based cohort study of 3,052 patients**

# Johanna Schönau, Axel Wester, Jörn M. Schattenberg*, Hannes Hagström*

# *Shared senior author

| Supplementary Table 1: ICD-10 codes used to define liver diseases and comorbidities | | |
| --- | --- | --- |
| Diagnosis | | **ICD-10** |
| PBC | | K743 |
| Liver transplantation | | Z94.4 |
| Alcoholic liver disease | | K70 |
| NAFLD | | K76.0, K75.8 |
| Viral hepatitis | | B15, B16, B17, B18, B19 |
| AAT deficiency | | E88.0A, E88.0B |
| Budd-Chiari | | I82.0, K765 |
| Hemochromatosis | | E831 |
| Wilson’s disease | | E83.0B |
| PSC | | K83.0 |
| AIH | | K75.4 |
| CVD | | I20-I25, I60-I69, I50, I110, I130, I132, I739, I74 |
| Diabetes | | E10, E11 |
| Cirrhosis | | K746 |
| IBD | | K50, K51 |
| COPD | | J44 |
| Reumatic disease | | Rheumatoid arthritis: M05, M06  Systemic inflammatory diseases of connective tissue/muscles: M30-M36 |
| Compensated Cirrhosis | | Cirrhosis coding: K746, K74,5  Or  Non-bleeding varices: I85.9, I98.2 |
| Decompensated cirrhosis | | Bleeding varices: I85.0, I98.3  Ascites: R18.9  Hepatorenal syndrome: K76.7 |
| Supplementary Table 2: ICD-10 codes for cancers | | |
|  | **ICD-10** | |
| Primary outcome |  | |
| Any cancer (except non-melanoma skin cancer) | C00-C97 except C44 | |
| Secondary outcomes |  | |
| Gastrointestinal | Esophageal: C15  Stomach: C16  Small intestine: C17  Colorectal C18-C21 | |
| HCC | C220 | |
| Pancreaticobiliary | Biliary: C221, C23, C24  Pancreas: C25 | |
| Lung | C34 | |
| Breast | C50 | |
| Gentourinary | Cervix: C53  Uterus: C54, C55  Ovary: C56  Prostate: C61  Kidney: C65  Urine bladder: C67 | |
| Lymphoma | C81-C88 | |
| Hematologícal cancers except lymphoma | Myeloma: C90  Leukemia and other hematological cancers: C91-C96 | |

| Supplementary Table 3:  ICD-10 codes for causes of death (main or contributing causes) from the Cause of Death Registry | |
| --- | --- |
|  | **ICD-10** |
| Cancer-related death | C00-C97 |
| Non-cancer-related death | Any death without the codes C00-C97 as either main or contributing causes |

| **Supplementary Table 4: Rate of any cancer in subgroups of patients with PBC and their reference individuals** | | | | | | |
| --- | --- | --- | --- | --- | --- | --- |
|  | **Patients with PBC**  **Events, n (%)** | **Reference individuals**  **Events, n (%)** | **Patients with PBC**  **Incidence rate/1000 PY (95% CI)** | **Reference individuals**  **Incidence rate/1000 PY (95% CI)** | **Unadjusted HR (95% CI) *** | **Adjusted HR (95% CI) **** |
| **Sex** |  |  |  |  |  |  |
| Women | 323 (12.4) | 2457 (10.7) | 18.0 (16.2-20.1) | 13.3 (12.8-13.9) | 1.4 (1.2-1.6) | 1.4 (1.2-1.5) |
| Men | 51 (11.4) | 474 (12.5) | 21.6 (16.4-28.5) | 18.0 (16.4-19.7) | 1.3 (0.9-1.7) | 1.3 (0.9-1.7) |
| **Age group** |  |  |  |  |  |  |
| <50 | 33 (7.3) | 205 (5.0) | 9.4 (6.7-13.3) | 5.9 (5.2-6.8) | 1.5 (1.1-2.2) | 1.4 (0.9-2.1) |
| 50-65 | 176 (14.2) | 1291 (11.5) | 18.2 (15.7-21.1) | 13.0 (12.3-13.7) | 1.5 (1.2-1.7) | 1.4 (1.2-1.7) |
| >65 | 165 (12.1) | 1435 (12.6) | 23.2 (19.9-27.0) | 18.8 (17.8-19.8) | 1.3 (1.1-1.5) | 1.3 (1.1-1.5) |
| **Disease severity *** |  |  |  |  |  |  |
| Cirrhosis | 35 (10.8) | 309 (11.4) | 27.6 (19.8-38.4) | 15.5 (13.9-17.4) | 2.0 (1.4-2.9) | 2.1 (1.4-3.0) |
| No cirrhosis | 339 (12.4) | 2622 (10.9) | 17.8 (16.0-19.8) | 13.7 (13.2-14.3) | 1.3 (1.2-1.5) | 1.3 (1.2-1.5) |

Abbreviations: PBC, primary biliary cholangitis; PY, Person years; HR, hazard ratio; HCC, hepatocellular carcinoma.

*Disease severity (cirrhosis/no cirrhosis) only refers to patients with PBC.

| **Supplementary Table 5: Mortality rate in patients with PBC and reference individuals diagnosed with non-HCC cancer during follow-up** | | | | | | |
| --- | --- | --- | --- | --- | --- | --- |
|  | **Patients with PBC**  **Events, n (%)** | **Reference individuals**  **Events, n (%)** | **Patients with PBC**  **Incidence rate/1000 PY (95% CI)** | **Reference individuals**  **Incidence rate/1000 PY (95% CI)** | **Unadjusted HR (95% CI)** | **Adjusted HR (95% CI)** |
| **1-year mortality** |  |  |  |  |  |  |
| All-cause | 73 (21.9) | 570 (19.6) | 269.2 (214.0-338.6) | 237.6 (218.9-258.0) | 1.3 (1.0-1.6) | 1.2 (0.9-1.6) |
| Cancer-related | 63 (18.9) | 524 (18.0) | 232.3 (181.5-297.4) | 218.5 (200.5-238.0) | 1.2 (0.9-1.6) | 1.1 (0.9-1.5) |
| Non-cancer-related | 10 (3.0) | 46 (1.6) | 36.9 (19.8-68.5) | 19.2 (14.4-25.6) | 2.2 (1.1-4.4) | 2.0 (1.0-4.1) |
| **5-year mortality** |  |  |  |  |  |  |
| All-cause | 132 (39.5) | 1002 (34.4) | 150.2 (126.6-178.1) | 123.5 (116.1-131.4) | 1.4 (1.1-1.6) | 1.3 (1.1-1.5) |
| Cancer-related | 103 (30.8) | 871 (29.9) | 117.2 (96.6-142.1) | 107 (100.4-114.7) | 1.2 (1.0-1.5) | 1.2 (0.9-1.4) |
| Non-cancer-related | 29 (8.7) | 131 (4.5) | 33.0 (22.9-47.5) | 16.1 (13.6-19.2) | 2.6 (1.7-3.9) | 2.2 (1.4-3.3) |

Abbreviations: PBC, primary biliary cholangitis; PY, Person years; HR, hazard ratio.
